# Empiric antibiotic therapy in acute uncomplicated urinary tract infections and fluoroquinolone resistance: a prospective observational study

**DOI:** 10.1186/1476-0711-8-27

**Published:** 2009-10-24

**Authors:** Cenk Aypak, Adalet Altunsoy, Nurşen Düzgün

**Affiliations:** 1 Department of Family Medicine, Ankara University, School of Medicine, Ibni Sina Hospital 06100, Ankara, Turkey; 2 Department of Clinical Microbiology and Infectious Disease, Ankara University, School of Medicine, Ibni Sina Hospital 06100, Ankara, Turkey; 3 Department of Clinical Immunology and Rheumatology, Ankara University, School of Medicine, Ibni Sina Hospital 06100, Ankara, Turkey

## Abstract

**Background:**

The aims of this study were to determine the antimicrobial susceptibility patterns of urinary isolates from community acquired acute uncomplicated urinary tract infections (uUTI) and to evaluate which antibiotics were empirically prescribed in the outpatient management of uUTI.

**Methods:**

Among the patients which were admitted to outpatient clinics of Ankara University Medical Faculty, Ibni-Sina Hospital during 2005-2006, a total of 429 women between the age of 18 and 65 years old who were clinically diagnosed with uUTI and to whom prescribed empirical antibiotics were enrolled in this prospective observational study. Patients' demographical data, urine culture results, resistance rates to antimicrobial agents and prescribed empiric antimicrobial therapy were analyzed.

**Results:**

Totally 390 (90.9%) patients among all study population were requested for urine culture by their physicians. 150 (38.5%) of these urine cultures were positive. The most common isolated uropathogen was *Escherichia coli (E. coli) *(71.3%). The variations of uropathogens according to age and menopause status were not significantly different.

The resistance rates of *E. coli *isolates for ampicillin, ampicillin-sulbactam, amoxicillin-clavulonate, cefuroxime, ceftriaxone, fluoroquinolones (FQ), co-trimoxazole (TMP-SMX) and gentamicin were 55.1%, 32.7%, 32.7%, 23.4%, 15.9%, 25.2%, 41.1%, 6.1% respectively. FQ were the most common prescribed antibiotics (77.9%) (*P *< 0.001), followed by TMP-SMX (10.7%), fosfomycin (9.2%), nitrofurantoin (2.1%). Treatment durations were statistically longer than the recommended 3-day course (*P *< 0.001).

**Conclusion:**

Empirical use of FQ in uUTI should be discouraged because of increased antimicrobial resistance rates.

## Background

Community acquired urinary tract infection (UTI) in women is a prevalent problem in primary care, accounting for approximately eight million ambulatory visits annually in the United States [1]. UTI has several different clinical presentations [1]. Acute uncomplicated UTI (uUTI) occurs in otherwise healthy non-pregnant women with normal genitourinary tract [2]. The current treatment of uUTI is empirical, based on the limited and predictable spectrum of etiological microorganisms [3]. However, as with many community acquired infections, resistance rates to antimicrobials which are commonly used in uUTI is increasing and susceptibility of microorganisms shows significant geographical variations [4-6]. The most important driving factor of resistance is overuse of antimicrobials [4-6]. Increasing antimicrobial resistance complicates uUTI treatment by increasing patient morbidity, costs of reassessment and re-treatment and use of broader spectrum antibiotics. Several studies have demonstrated increasing antibiotic resistance levels in *E. coli *causing community acquired UTI but most *in vitro *data come from laboratory based surveys that often do not define the sex, age, clinical syndrome or other data of interest regarding the patients from whom the urine specimens were collected [7]. And also even in the same country the susceptibility patterns of the microorganisms exhibit regional differences [8]. Appropriate knowledge about local and national antimicrobial resistance trends is the utmost importance in order to setup evidence based recommendations in empirical antibiotic treatment of uUTI [8,9].

We therefore in this prospective observational study aimed to obtain data on the resistance rates of common pathogens in 18-65 years old female patients with uUTI and to determine which empiric antibiotics are prescribed in relevant settings at our university hospital for the outpatient management of community acquired uUTI.

## Materials and methods

### Data collection and Patients

Female patients aged between 18-65 years who attended to a tertiary care hospital emergency department and outpatient clinics (Ankara University Medical Faculty Ibni-Sina Hospital Ankara, Turkey which has 1000 beds and admits more than 30 000 patients in emergency department annually) during the period between 1 March 2005 and 1 September 2006, with symptoms of community acquired acute uUTI and to whom empiric antibacterial treatment had prescribed were enrolled in this prospective observational study.

The symptomatic uUTI described by set of symptoms involving dysuria, frequency, urgency and suprapubic tenderness without the presence of fever. Diagnosis were made and recorded by the treating physician. Exclusion criteria were symptoms for > 7 days, signs of pyelonephritis (a body temperature > 38°C orally, flank pain or costovertebral angle tenderness), three or more episodes of UTI in the past year, symptoms of UTI in the last three months, previous upper UTI, other functional and structural urinary tract abnormalities, indwelling or recent Foley catheter, previous history of genitourinary system operation including urinary stones, current pregnancy, antibiotic use during the previous three months, patients who were hospitalised for any reason during past three months, patients with diabetes mellitus and known immune compromised state.

Patients' demographical data, symptoms, physical examination results, urinalysis, urine culture results, pathogen microorganisms and resistance rates to antimicrobials and prescribed empiric antimicrobial therapy (agent and duration) were recorded by a trained physician or a nurse.

### Laboratory Methods

The urine specimens were taken after instructing the patient on the midstream technique. Pyuria was detected either with positive dip-stick test or > 5-10 leucocytes in the urine centrifuged at 2000 rpm for 5 minutes. Clean-catch urine samples obtained from patients were inoculated onto 5% blood agar and Eosin-Methylene Blue (EMB) agar with 0.01 mL calibrated loops by a semi-quantitative technique. Culture plates were incubated for 18-24 h at 37°C. A threshold of > 10^5 ^organisms per mL of urine defined as a positive culture. The isolated bacteria were identified by conventional methods and BBL Crystal Enteric/NF 4.0 identification kits (Becton Dickinson-NY, USA) were used when needed. The susceptibility of each isolated pathogen to antibiotics (the fluoroquinolones (FQ), ampicillin, ampicillin-sulbactam, amoxicillin-clavulonate, co-trimoxazole (TMP-SMX), ceftriaxone, cefuroxime, gentamicin) were determined by the Kirby-Bauer disc diffusion method and by automatic system (Becton Dickinson). Samples were processed in the University Hospital Laboratory according to standard procedures defined by the Clinical and Laboratory Standards Institute (CLSI, formerly the National Committee for Clinical Laboratory) [10].

The hard paper copies of locally generated reports and susceptibility results were collected and added to the patients' records.

### Statistical analysis

The data from the study were inserted into MS Excel, coded than transferred into SPSS 14.0 for Windows for statistical analysis. Pearson chi-squared test was used to compare the parameters. Data are presented with 95% confidence intervals (CI). A *P *value of < 0.05 was considered statistically significant.

### Ethics

Ethical approval was granted for this study by Ankara University Local Research Ethics Committee (No: 73-1877)

## Results

### Patient demographics

A total of 429 female patients were diagnosed with uUTI and received empiric antibiotics included in the study. 78.3% of the patients (336) were enrolled from emergency department, 16.6% of patients (71) from internal medicine and, 5.1% (22) from urology outpatient clinics. 278 of them (64.8%) were belonged to the age group of 18-50 years. 151 (35.2%) patients were between 50 and 65 years of age. Mean age of the study population was 42.41 (± 14.7). 276 (64.3%) of the patients were premenopausal and 153 (35.7%) were postmenopausal.

### Isolated uropathogens

Urine culture was ordered for 390 (90.9%; 95% CI 88.1-93.6%) of the study population by their physicians consequently and 150 (38.5%; 95% CI 33.5-43.4%) of these cultures were positive for bacterial growth. The remaining 240 specimens (61.5%; 95% CI 56.6-66.4%) were negative for bacterial growth. 39 (9.1%; 95% CI 6.3-11.8%) patients received antibiotics without the request of urine culture. Gram-negative bacteria were found in 115 (76.7%; 95% CI 69.7-83.6%) urines and gram-positive bacteria in 24 (16%; 95% CI 10-22%), *Candida *spp. in 11 (7.3%; 95% CI 3.1-11.5%). The distribution of uropathogen groups according to menopausal status were not significantly different (Table 1).

**Table 1:** The distribution of microorganisms according to menopausal status

**Microorganism**	**Premenopause n (%)**	**Postmenopause n (%)**	***P *value**
*Gram (-)bacteria*	81 (80.2%)	34 (69.4%)	0,142
*Gram (+)bacteria*	14 (13.9%)	10 (20.4%)	0.305
*Candida *spp.	6 (5.9%)	5 (10.2%)	0.348

***Total***	**101 (% 100)**	**49 (% 100)**	

The most common isolated uropathogen was *E. coli *(71.3%; 95% CI 63.9-78.6%), followed by *Staphylococcus *spp. (8%; 95% CI 3.6-12.4%), *Candida *spp. (7.3%; 95% CI 3.1-11.5%), *Streptococcus *spp. (4.7%; 95% CI 1.2-8.2%), *Klebsiella *spp. (4%; 95% CI 0.8-7.2%), *Enterococcus *spp. (3.3%; 95% CI 0.2-6.3%), *Proteus *spp. (1.3%; 95% CI 0.6-3.2%) were isolated respectively.

### Antimicrobial resistance patterns

The antibiotic resistance rates of the isolates are shown in table 2. The resistance rates of most prevailing microorganism, *E. coli *isolates for ampicillin, ampicillin-sulbactam, amoxicillin-clavulonate, cefuroxime, ceftriaxone, FQ, TMP-SMX and gentamicin were 55.1% (95% CI 45.4-64.7%), 32.7% (95% CI 23.6-41.7%), 32.7% (95% CI 23.6-41.7%), 23.4% (95% CI 15.2-31.6%), 15.9% (95% CI 8.8-22.9%), 25.2% (95% CI 16.8-33.6%), 41.1% (95% CI 31.6-50.6%), 6.1% (95% CI 4.4-8.4.7%) respectively. Resistance to TMP-SMX (41.1%; 95% CI 31.6-50.6%) was found to be higher than FQ resistance (25.2% 95% CI 16.8-33.6%). FQ+TMP-SMX dual resistance was 17.8% (95% CI 10.4-25.2%) among the *E. coli *isolates. Although there was a tendency in increase of resistance rates to all beta-lactam antibiotics in patients older than fifty years of age, only cefuroxime resistance in *E. coli *isolates according the age group of patients was statistically significant (17.3% versus 37.5%; *P *< 0.05), (Table 3).

**Table 2:** Isolated bacteria and their resistance rates to antimicrobial agents in 139* urine samples

**Bacterium % resistance** **(n, %)**	**Ampicillin**	**Ampicillin-** **Sulbactam**	**Cefuroxime**	**Ceftriaxone**	**Amoxicillin-clavulonate**	**Fluoroquinolones**	**Gentamicin**	**TMP-SMX**^§^
***E. coli*** **% resistance** (107, 71.3%)	55.1	32.7	23.4	15.9	32.7	25.2	6.1	41.1
***Staphylococcus *spp**. **% resistance **(12, 8%)	_**	41.7	41.7	_**	41.7	41.7	41.7	33.3
***Streptococcus *spp.** **% resistance **(7, 4.7%)	0	0	0	0	0	0	_**	_**
***Klebsiella *spp.** **% resistance **(6, 4%)	100	16.7	16,7	0	16,7	0	16.7	16,7
***Proteus *spp**. **% resistance **(2, 1.3%)	0	0	0	0	0	0	0	0
***Enterococcus *spp**. **% resistance **(5, 3.3%)	0	_**	_**	_**	_**	20	40	_**

* Candida isolates were not included; n = number of isolates; ** = not determined; ^§ ^= co-trimoxazole

**Table 3:** Resistance rates of *E. coli *strains according the age groups

**Antimicrobial Agent**	**Age < 50**	**Age ≥ 50**	***P *value**
Ampicillin	54.7%	56.3%	0.880
Ampicillin-sulbactam	29.3%	40.6%	0.254
Amoxicillin-clavulonate	29.3%	40.6%	0.254
Cefuroxime	17.3%	37.5%	0.024*
Ceftriaxone	13.3%	21.9%	0.268
Fluoroquinolone	22.7%	31.3%	0.349
Gentamicin	5.8%	6.3%	0.585
TMP-SMX**	46.7%	28.1%	0.074
TMP-SMX+FQ***	18.7%	15.6%	0.706

* = *P *< 0.05; ** = co-trimoxazole; *** = co-trimoxazole, fluoroquinolone dual resistance

### Prescribed antimicrobials and treatment durations

**Figure 1:**
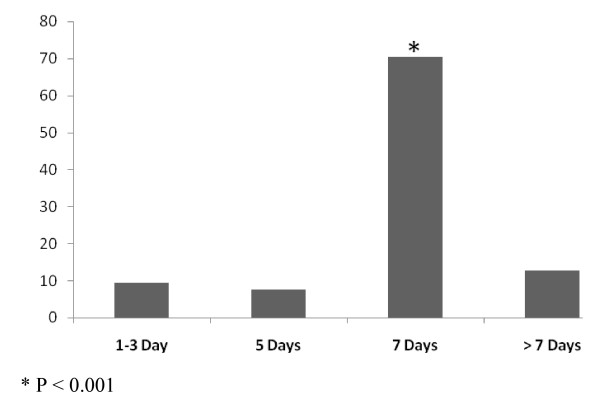
Antimicrobial treatment durations (%) * P < 0.001.

For 429 patients with uUTI, FQ were the most common empirically prescribed antibiotics (77.9%; 95% CI 73.9-82.1%) by the physicians compared to TMP-SMX (10.7%; 95% CI 7.7-13.7%), fosfomycin (9.2%; 95% CI 6.4-12%), nitrofurantoin (2.1%; 95% CI 0.7-3.5%) (*P *< 0.001). The first choice among FQ was ciprofloxacin (82.7%; 95% CI 78.6-86.8%) followed by ofloxacin (7.6%; 95% CI 4.7-10.5%) levofloxacin (7.3%; 95% CI 4.5-10.1) and moxifloxacin (2.4%%; 95% CI 0.7-4.1%), (Table 4). Treatment durations were statistically longer than the recommended 3-day course; 1-3 day (9.4%; 95% CI 6.6-12.2%), 5 days (7.5%; 95% CI 4.9-10%), 7 days (70.4%; 95% CI 65.9-74.8%), > 7 days (12.7%; 95% CI 9.5-15.9%) (*P *< 0.001), (Figure 1). Prescription rates of the different classes of drugs and duration of treatment were not statistically significant between age groups (*P *= 0.469 and *P *= 0.615 respectively).

**Table 4:** Empirically prescribed antimicrobial agents

**Antimicrobial Agent**	**n**	**%**
**Fluoroquinolone**	334	77.9%
Ciprofloxacin	277	82.7%
Ofloxacin	25	7.6%
Levofloxacin	24	7.3%
Moxifloxacin	8	2.4%
TMP-SMX*	46	10.7%
**Fosfomycin**	40	9.2%
**Nitrofurantoin**	9	2.1%

Total	429	100%

* = co-trimoxazole

## Discussion

This study shows the distribution of microbial species isolated from patients with uUTI and their resistance rates to antimicrobial agents at a university hospital in Turkey.

As it has previously been reported, we found the majority of patients with uUTI were under 50 years of age and the predominant bacterium was *E. coli *[11]. The uropathogens isolated in this patient population were similar to those in other comparable studies [12,13]. It was reported that in postmenopausal women owing to the loss of oestrogen and consequent changes in vaginal flora, the etiological agents in uUTI can be different from premenopausal patients [14-16]. Our data have not revealed such a difference between these two groups of patients.

Main international guidelines recommend empirical therapy in uUTI [17,18]. The efficacy of such empirical therapy depends on periodic assessment of antimicrobial resistance profiles. Although the spectrum of bacteria isolated from patients with uUTI worldwide has remained largely unchanged in which *E. coli *is the most prevalent microorganism, there have been significant changes in the resistance patterns of uropathogens over the past few decades and antibiotic resistance has become a major problem in uUTI [13]. Increasing antimicrobial resistance has been documented all over the world [11,19,20]. Resistance rates among strains of *E. coli *isolated from women with uUTI averages 30% for both sulphonamides and ampicillin, varying from 17% to 54% in different countries [11]. Trimethoprim resistance ranges from 11% in Scandinavian countries to 34% in Spain and Portugal. FQ resistance was not found in Scandinavian countries but reaches 20% in southern Europe [11]. Recently Gobernado *et al*. showed reduced susceptibility of *E. coli *strains isolated from patients with uUTI to TMP-SMX (26%) and to FQ (16%) in Spain where antimicrobials can be used without restriction [8]. The variability amongst different centres confirms the need for local resistance prevalence data to be available to the practitioner who treats UTI, especially where empirical therapy is being used for urinary infections.

Our study clearly shows that there is a significant increase in TMP-SMX and FQ resistance among *E. coli *isolates from patients with uUTI in our region which makes the empirical treatment of uUTI a great challenge. This observation is in accordance with recent studies conducted in Spain and Europe [8,11]. Arslan *et al*. reported 36% resistance to TMP-SMX and 17% resistance rates to FQ among 288 *E. coli *isolates from patients with uUTI in Turkey [21]. Also Ozyurt *et al*. have found 34% resistance to TMP-SMX and 18% resistance to FQ among community-acquired uropathogen *E. coli *isolates from Istanbul region [22]. The reported rates of resistance among uropathogens may vary depending on whether study sample consist of primarily of outpatients with uUTI or patients with complicated UTI.

Influence of age has previously been shown to have an impact on resistance rates in UTI [20,21,23,24]. Although our study population was younger than comparable studies, *E. coli *isolates of this study were also more likely to be resistant to ampicillin, ampicillin-sulbactam, amoxicillin-clavulonate, ceftriaxone, gentamicin, and FQ in the patients over fifty years of age. Cefuroxime resistance is the only statistically significant one.

In several studies it has been shown that the prescribing habits of the physicians are the driving factor for the antibiotic resistance [25-27]. Goettsche *et al*. reported that the resistance against FQ is strongly associated with a high number of prescriptions for this group of antibiotics [25]. Because of aforementioned reasons pharmacological surveillance is an essential part of antimicrobial agent resistance studies. Although the association between antimicrobial agent utilisation and resistance in hospital services is well known, there is a lack of information for the same issue in community acquired infections [28-30].

There are relatively few studies published on variations in treatment for uUTI. McEwen *et al*. found that 37% of physicians actually prescribe TMP-SMX, closely followed by FQ (32%) and average duration of antibiotic therapy was 8.6 days in the United States [31]. Kahan NR *et al*. revealed that FQ were the most frequently prescribed drugs (25.57%) in Israel [32]. In our study we found that although not recommended as first line antibiotic, FQ were the most frequent empirically prescribed drugs in our hospital settings for uUTI. In the FQ group, ciprofloxacin was the most commonly prescribed drug and the average duration of the therapy was longer than the recommended 3 days. The prescribed antimicrobial agent and duration of the treatment were not different between age groups.

To our knowledge this is the first study in Turkey which directly evaluates the prescription behaviour of treating physicians on a medical condition. The data have not been collected from a drug surveillance database or from medical records retrospectively without knowledge of patient's clinical situation. The results are based on the actual physician habits, so it does give an accurate description of which antibiotics are prescribed and the duration of therapy. Since uUTI is relatively common, widespread inappropriate prescribing increases resistance among uropathogens. Our resistance rates to FQ among *E. coli *strains were found to be much higher than other European studies. This may be due to high use of FQ, since it is considered the antimicrobial group of choice in UTI. Inappropriate antibiotic prescribing for UTI was documented in 47.3% of patients in a study from Turkey [33]. In addition to increasing risk of resistance; current prescribing patterns in our hospital increase the medical costs. In this study we also found that newer FQ like moxifloxacin and levofloxacin were also prescribed for the treatment of uUTI. It is known that among susceptible isolates, the more expensive broad-spectrum FQ are not more effective than the cheaper alternatives [1,12].

### Limitations

Although it is local, we believe that in this observational prospective study we reached our goal in terms of obtaining precise scientific data dealing with the resistance rates of uropathogens in a university hospital serving to the central Anatolia region. At the same time we had an opportunity to evaluate the actual prescribing habits of our physicians in a medical condition which is most of the times improperly treated.

We did not have a chance to evaluate the resistance patterns of *E. coli *isolates to the other alternative antimicrobials such as nitrofurantoin and fosfomycin due to the lack of antimicrobial discs in our hospital laboratory.

Further studies with larger number of isolates from each individual geographical region are needed to confirm our results. However clinicians should be aware of regional resistance rates and it should be taken into consideration before initiating empirical antimicrobial therapy for uUTI.

## Conclusion

FQ should be used with consideration since the resistance to FQ is in increase; rather it ought to be prescribed for treatment of serious infections like connective tissue infections, respiratory tract infections and upper UTI. If conditions allowing use of nonfluoroquinolone drugs for the treatment of uUTI should be encouraged when TMP-SMX is not an option. Since uUTI is relatively easy to cure with limited morbidity, agents like nitrofurantoin and fosfomycin should be used instead of FQ.

## Competing interests

The authors declare that they have no competing interests.

## Authors' contributions

CA has collected the data with the collaboration of AA. CA has performed all the analysis incorporated in this manuscript. ND has designed the problem and guide throughout this study as well as helped in writing this manuscript. CA has written first draft of manuscript.
